# Comparing linear and non-linear models to estimate the appropriate cochlear implant electrode array length—are current methods precise enough?

**DOI:** 10.1007/s00405-023-08064-z

**Published:** 2023-07-19

**Authors:** Nora M. Weiss, Tabita Breitsprecher, Martin Wozniak, David Bächinger, Christiane Völter, Robert Mlynski, Paul Van de Heyning, Vincent Van Rompaey, Stefan Dazert

**Affiliations:** 1 Department of Otorhinolaryngology-Head and Neck Surgery, Ruhr-University Bochum, St. Elisabeth-Hospital Bochum, Bochum, Germany; 2 Department of Translational Neurosciences, Faculty of Medicine and Health Sciences, University of Antwerp, Antwerp, Belgium; 3 International Graduate School of Neuroscience (IGSN), Ruhr-University Bochum, Bochum, Germany; 4 MED-EL Elektromedizinische Geräte Deutschland GmbH, Starnberg, Deutschland; 5 Department of Otorhinolaryngology, Head and Neck Surgery, “Otto Körner”, University, Rostock, Germany; 6 Department of Otorhinolaryngology and Head & Neck Surgery, Antwerp University Hospital, Antwerp, Belgium

**Keywords:** Angular insertion depth prediction, Insertion angle

## Abstract

**Purpose:**

In cochlear implantation with flexible lateral wall electrode arrays, a cochlear coverage (CC) range between 70% and 80% is considered ideal for optimal speech perception. To achieve this CC, the cochlear implant (CI) electrode array has to be chosen according to the individual cochlear duct length (CDL). Here, we mathematically analyzed the suitability of different flexible lateral wall electrode array lengths covering between 70% and 80% of the CDL.

**Methods:**

In a retrospective cross-sectional study preoperative high-resolution computed tomography (HRCT) from patients undergoing cochlear implantation was investigated. The CDL was estimated using an otosurgical planning software and the CI electrode array lengths covering 70–80% of the CDL was calculated using (i) linear and (ii) non-linear models.

**Results:**

The analysis of 120 HRCT data sets showed significantly different model-dependent CDL. Significant differences between the CC of 70% assessed from linear and non-linear models (mean difference: 2.5 mm, *p* < 0.001) and the CC of 80% assessed from linear and non-linear models (mean difference: 1.5 mm, *p* < 0.001) were found. In up to 25% of the patients none of the existing flexible lateral wall electrode arrays fit into this range. In 59 cases (49,2%) the models did not agree on the suitable electrode arrays.

**Conclusions:**

The CC varies depending on the underlying CDL approximation, which critically influences electrode array choice. Based on the literature, we hypothesize that the non-linear method systematically overestimates the CC and may lead to rather too short electrode array choices. Future studies need to assess the accuracy of the individual mathematical models.

## Introduction

Cochlear implantation is the therapy of choice for patients with severe to profound hearing loss that do not benefit from conventional hearing aids [1]. Determining suitable cochlear implant (CI) electrode array lengths according to the individual cochlear anatomy is a matter of debate. In studies reporting advantageous effects from deeper insertion angles, it is hypothesized that a deeper insertion of the CI electrode array into the apical region of the cochlea may enhance speech perception outcomes due to an improved match between programmed frequency bands of the electrode array and the tonotopic organization of the cochlea [2–4]. Reasons for a potential poorer performance caused by a deeper electrode array insertion may be due to apical frequency pitch confusions caused by the closer contact between the portions of the electrode array, or an increased damage to the cochlea due to the insertion trauma [5, 6]. Apart from residual hearing and stimulation strategy (electroacoustic versus electric-only), the electrode array choice may also be based on individual preferences of the surgeon. For these reasons, one of the CI manufacturers (Med-El, Innsbruck, Austria) produces flexible lateral wall electrode arrays in different lengths [7]. Using these flexible lateral wall electrode arrays, Canfarotta et al. found significantly better speech performance in patients with longer electrode arrays at a mean angular insertion depth (AID) of 628° compared to those with an AID of 571°. A plateau in performance was observed in AIDs around 600° [8]. Another study reporting a beneficial effect of deeper electrode array insertion with flexible lateral wall electrode arrays reports insertion depths beyond 70% of the cochlear duct length (CDL) [9], whereas a study with 56 patients with a mean AID of 90% showed no association between the insertion depth and the speech performance [10]. Thus, when an individualized electrode array choice according to the estimated CDL with flexible lateral wall electrode arrays is chosen, an ideal insertion depth between 70% and 80% is often recommended [11]. This corresponds to the neural apex of the spiral ganglion between 650° and 690° reported in previous studies [12, 13]. Deeper insertions are even considered deleterious [14]. However, to achieve an ideal cochlear coverage (CC), the CI electrode array has to be chosen according to the size of the cochlea. Estimating the CDL from preoperative imaging to assess the respective electrode array length is time consuming and may underlie interobserver variances [15]. On the other hand, the choice of the electrode array length may influence structure preservation and thus hearing preservation [16].

In this study, it is hypothesized that a CC range between 70% and 80% is achieved by two different electrode array sizes in a majority of patients. Furthermore, the CC range may vary depending on the underlying calculation method (linear versus non-linear). Therefore, in this study, we aimed to investigate the number of single flexible lateral wall electrode arrays covering 70–80% of the CDL of individual patients undergoing cochlear implantation. It is further hypothesized that the non-linear method systematically overestimates the CC and may lead to rather too short electrode array choices.

## Methods

In this single center retrospective cross-sectional study, CT imaging from patients that received a CI between January 2020 and September 2022 due to severe or profound hearing loss were analyzed. The CT slice thickness varied between 0.625 mm and 1 mm. This study was designed in accordance with the Declaration of Helsinki and its amendments. The CDL was estimated by different methods as detailed below and the suitable electrode array length covering 70–80% of the CDL was determined.

### CDL estimation

The surgical planning software “Otoplan” (Version 2.0, Cascination AG, Bern, Switzerland) was used to estimate the CDL and insertion depth of the different electrode arrays. The surgical planning software estimates the CDL from preoperative CT imaging data. Depending on the estimated size of the cochlea, the software indicates the respective CC by the different electrode array lengths. The software requires the user to define the diameter (i.e., the *A value*) and width of the cochlea basal turn in the oblique coronal view (i.e., the *B value*, which is defined as the cochlear width perpendicular to the line segment of the *A value*, intersection point modiolus). The CDL calculation is based on the elliptic circular approximation and percentage of basal turn length as reported by Schurzig et al. [17]. It may be calculated at specific angles (θ) in which θ is the angular depth along the cochlea in degree. By definition, the full CDL covering 900° along the lateral wall (CDL_LW_) is extrapolated from the basal turn length. Accordingly, CDL_LW_ is calculated as detailed below (Eq. 1).

1 $${\text{CDL}}_{LW} = 1.797 \times \left[ {1.18 \times A - {\text{value}} + 2.69 \times B - {\text{value }} - \sqrt {\left( {0.72 \times A - {\text{value}} \times B - {\text{value}}} \right)} } \right]$$

Newer software versions additionally estimate the CDL along the organ of Corti, which considers also the hook region (length of basilar membrane prior the centre of the round window membrane) using a correction factor of 0,5 mm for the *A-* and *B value* (Eq. 2) [19]:

2 $${\text{CDL}}_{OC} = \left[ {1.71 \times \left[ {1.18 \times \left( {A - {\text{value}}_{OC} } \right) + 2,69 \times \left( {B - {\text{value}}_{OC} } \right) - \sqrt {0.72 \times A - {\text{value}}_{OC} \times B - {\text{value}}_{OC} } } \right] 0.18} \right] + 1.57$$

### Electrode array visualization tool

The electrode array visualization tool uses the reciprocal value of Eq. 2 to calculate the angular insertion depth from the linear insertion depth. The distance from the electrode array stopper to the most apical electrode “Channel 1” (C1_linear_) is used and the cochlear coverage is calculated based on the CDL and a certain angle (Eq. 3):

3 $$pBTL\left( \theta \right) = \frac{{C1_{{{\text{linear}}}} }}{{\left[ {(1.18 \times \left( {A - {\text{value}} - 0.7} \right) + 2.69 \times \left( {B - {\text{value}} - 0.7} \right) - \sqrt {(0.72 \times \left( {A - {\text{value}} - 0.7} \right) \times \left( {B - {\text{value}} - 0.7} \right)} } \right]}}$$

The different lateral wall electrode arrays can then be visualized based on the individual anatomy, which aids the surgeon in choosing an appropriate electrode array.

### Electrode array estimation

Flexible lateral wall electrode arrays from the CI manufacturer Med-El were chosen as the basis for the analysis, because they are designed for an individualized size approximation and are available in different lengths. Med-El is the only manufacturer offering different electrode array lengths of the same electrode array design (flexible lateral wall electrode arrays). Lengths of 20 mm (Flex20 electrode array, Med-El), 24 mm (Flex24 electrode array, Med-El), 26 mm (Flex26 electrode array, Med-El), 28 mm (Flex28 electrode array, Med-El) and 31.5 mm (FlexSoft electrode array, Med-El) are available. The electrode array designs covering 70–80% were calculated according to the estimated CDL by.

1. Linear model (mm; linear insertion depth [LID]) using Eq. 1 (CC_LID-70%_ and CC_LID-80%_)
2. Non-linear calculation (mm; obtained from the angular insertion depth [AID]) using Eq. 3 (CC_AID-630°_and CC_AID-720°_)

Since the electrode array is designed to lie along the lateral wall, the CDL_LW_ was used as the basis for the linear model.

### Statistical analysis

Statistical analyses were performed using Microsoft Excel (version 15.29, Microsoft Corporation) and Prism (version 8, GraphPad Software). The significance level was set to *p* < 0.05. The assumption of normality was tested graphically using quantile–quantile plots. Differences between two groups were assessed using Student’s *t* test. Data are presented as mean with standard deviation (SD) as well as absolute numbers with percentages.

## Results

**Fig. 1:**
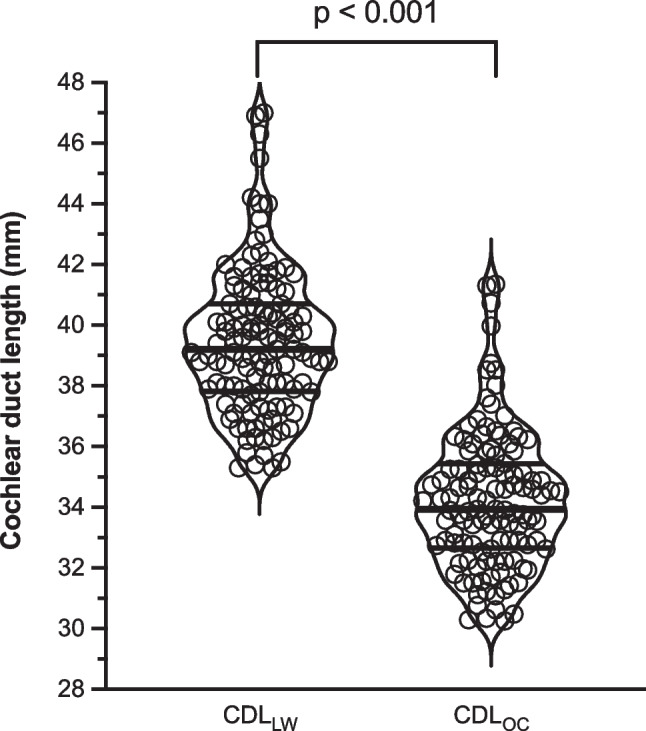
Violin plot showing the distribution of cochlear duct lengths (CDL). CDL assessed at the lateral wall (CDL_LW_) and at the organ of corti (CDL_OC_). The shape of the “violin” represents a 90° rotated smoothed probability density plot of the data at different values, the horizontal lines indicate the 25% quartile, median and 75% quartile

A total of 120 HRCT imaging data sets from 104 patients were analyzed. The mean CDL assessed at the organ of corti (CDL_OC_) was 34.2 mm (SD 2.3 mm), which was significantly smaller than the mean CDL assessed at the lateral wall CDL_LW_ that was 39.5 mm (SD 2.4 mm; mean difference 5.2 mm; *p* < 0.001; Fig. 1).

**Fig. 2:**
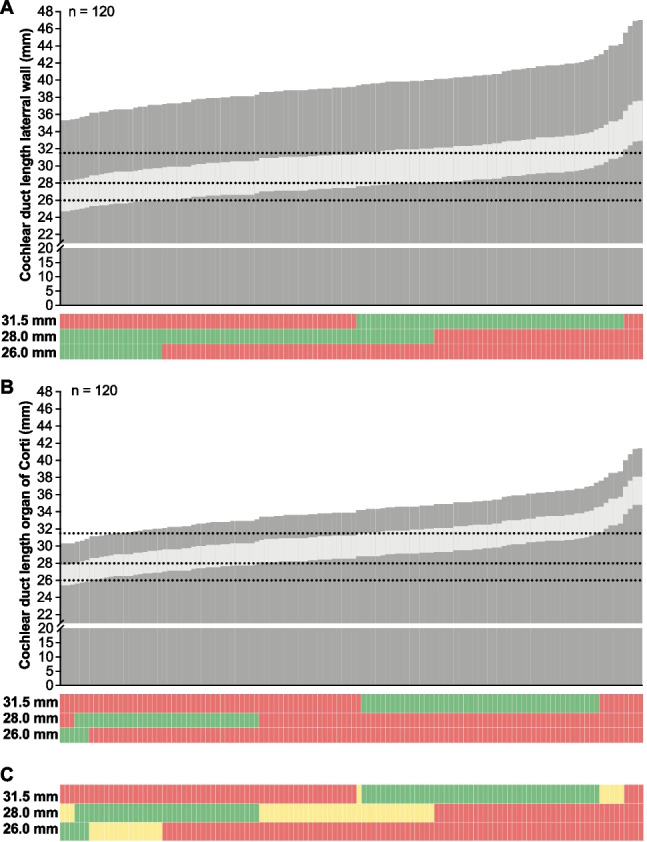
Individual cochlear duct lengths (CDL) and suitability for specific electrode array lengths. CDL assessed at the lateral wall (**A**) or at the organ of corti (**B**) for individual cochleae (represented by bars). The light grey section of the bar indicates a linear insertion depth between 70% and 80% cochlear coverage (CC) based on linear (**A**) and non-linear (**B**) models. Dotted lines indicate the linear insertion depth of a 26 mm, a 28 mm and a 31.5 mm electrode array. Below, color-coded bars indicate the suitability of specific electrode array lengths based on a 70% and 80% CC (red: not suited, green: suited). **C** Agreement between linear and non-linear model of suitable electrode array lengths (green: both models estimate electrode array as suitable; yellow: models do not agree on suitability of estimated electrode array; red: both models estimate electrode array as not suitable)

Assessing the LID, in 37 cases (30.8%) more than one electrode array length was eligible to cover 70–80% of the CDL, and in 4 cases (3.3%) none of the available electrode arrays fell within this range, because 70% could not be reached by any of the electrode arrays even the 31.5 mm electrode array. The 26 mm electrode array was calculated to cover 70–80% of the CDL in 6 out of 120 cases (5.0%), the 28 mm electrode array in 38 cases (31.7%) and the 31.5 mm electrode array in 49 cases (40.8%) (Fig. 2A).

Assessing the AID, in 3 cases (2.5%) more than one electrode array length was eligible to cover 630° to 720° of the CDL, in 30 cases (25.0%) none of the available electrode arrays fell within this range. In 9 of these cases 70% could not be reached by any of the electrode arrays even the 31.5 mm electrode array. In 21 of these cases the range fell between the 28 mm and the 31.5 mm electrode array (Fig. 2). The 26 mm electrode array was calculated to cover 70–80% of the CDL in 21 out of 120 cases (17.5%), the 28 mm electrode array in 77 cases (64.2%) and the 31.5 mm electrode array in 55 cases (45.8%) (Fig. 2B).

In 9 out of 120 cases (7.5%) assessing the AID and in 4 out of 120 cases (3.3%) assessing the LID, none of the existing electrode arrays was calculated to cover the range of 70–80% of the LID and 70% (i.e., 630°) to 80% (i.e., 720°) of the AID, respectively.

**Fig. 3:**
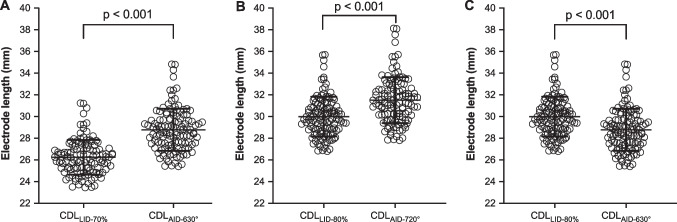
Scatterplot showing the individually calculated electrode array lengths. Significant differences between a cochlear coverage (CC) of 70% assessed from the cochlear duct length (CDL) assessed at the lateral wall (CDL_LW_) (CDL_LID-70%_) and an angular insertion depth (AID) of 630° assessed from the CDL assessed at the organ of corti (CDL_OC_) (CDL_AID-630°_) (mean difference: 2.5; *p* < 0.01) (**A**), between a cochlear coverage (CC) of 80% assessed from the cochlear duct length (CDL) assessed at the lateral wall (CDL_LW_) (CDL_LID-80%_) and an angular insertion depth (AID) of 720° assessed from the CDL assessed at the organ of corti (CDL_OC_) (CDL_AID-720°_) (mean difference: 1.5; *p* < 0.01) (**B**) as well as between a cochlear coverage (CC) of 80% assessed from the cochlear duct length (CDL) assessed at the lateral wall (CDL_LW_) (CDL_LID-80%_) and an angular insertion depth (AID) of 630° assessed from the CDL assessed at the organ of corti (CDL_OC_) (CDL_AID-630°_) (mean difference: 1.2; *p* < 0.01) (**C**) are shown. Bar indicates mean, whiskers indicate standard deviation

Comparing a CC of 70% assessed from the linear model (CC_LID-70%_) and from the non-linear model (CC_AID-630°_), a significant difference of 2.5 mm (SD 0.2 mm; *p* < 0.001; Fig. 3A) was found. The difference between a CC of 80% assessed from the linear model (CC_LID-80%_) and from the non-linear model (CC_AID-720°_) was 1.5 mm (SD 0.3 mm, Fig. 3B). When comparing the CC of 70% assessed from the non-linear model (CC_AID-630°_) with CC of 80% assessed from the linear model (CC_LID-80%_), the means converge but still show a significant difference (mean difference: 1.2 mm, SD 0.2 mm; *p* < 0.001; Fig. 3C).

## Discussion

In this study, we found that the calculated CC shows large variations depending on the underlying CDL approximation. This discrepancy seems to be due to the underlying models. The linear estimation model is based on the CDL_LW_ calculation based on the *A* and *B* value determination assessed from the otosurgical planning software (Eq. 1). In contrast, the software-integrated visualization tool displays the insertion angle along the organ of Corti, though the mathematical calculation basis for the AID is the CDL_LW_ (under consideration of the distance between the electrode array and the lateral wall; Eq. 3). Thus, the electrode array’s position in this tool is assumed to be positioned between the organ of Corti and the lateral wall. Furthermore, the visualization tool calculates with C1 instead of the electrode array’s silicone tip, which may also favor inaccuracies in the performed analysis. Thus, we hypothesize that both methods may systematically overestimate the CC and may lead to rather too short electrode array choices. The largest overlap between the underlying approximation could be shown for the 31.5 mm electrode array. However, in up to 25% of the patients none of the existing flexible lateral wall electrode arrays optimally fit into this range. Depending on the underlying CDL approximation, in 3–31% of the patients more than one flexible lateral wall electrode array length is eligible to cover the range between 70% and 80% LID as well as between 630° and 720° AID. A 28 mm electrode array covers this range in up to 64%, a 31.5 mm electrode array in up to 46% of the patients. However, in up to 25% the range between 70% and 80% is not covered by any of the available electrode arrays (Fig. 2C). The smaller the CDL is estimated, the smaller is the range between 70% and 80% leading to fewer considerable electrode array choices (Fig. 2B).

Understanding the variability in speech perception outcomes following cochlear implantation remains a challenge to researchers and clinicians. The insertion depth has been shown to account for up to about 30% of variability in speech perception in straight electrode arrays [9]. The insertion depth is affected by array length, array design, surgical approach and cochlear morphology. An explanation to be discussed why deeper insertions may benefit speech perception is an improved match between the tonotopy of the cochlea and the electric stimulation by the electrode array [2, 8, 19–28]. A greater insertion depth is assumed to cover a greater number of spiral ganglion cells [4, 13]. There is evidence for a positive correlation between insertion depth and speech perception in studies with lateral wall electrode arrays [24]. However, this effect seems to be of importance particularly within a range of 70–80% CC [11, 14].

The present study shows that in the majority of cases the goal of covering 70–80% of the CDL is achievable with the 28 mm electrode array or the 31.5 mm electrode array. Thus, the underlying estimation of the CDL plays an important role when a CC within this range is aimed resulting in the need for studies assessing the accuracy of CC prediction. In cases where more than one electrode array length is eligible to cover this range, the question of whether to choose the longer or the shorter electrode array is often answered according to the surgeon’s preference. Inserting the shorter electrode array may be technically easier and may lead to an improved structure preservation, e.g., in hearing preservation cases, without negative effects on the postoperative speech performance. In contrast, the longer electrode array may be preferred in intermediate sizes aiming at a higher CC. However, our data provide evidence that a 28 mm electrode array or a 31.5 mm electrode array is well-suitable to cover 70–80% of the CDL in the majority of patients with a trend towards even too small CCs when based on the linear estimation model. A recent review showed that a good hearing preservation is achievable with a 28 mm flexible lateral wall electrode array encouraging the use of longer electrode arrays in inconclusive cases [29]. Especially in areas with limited resources concerning presurgical planning software or high-resolution imaging, the information of this study may be of interest. In cases where the examiner or surgeon is indecisive between two electrode array lengths, the shorter electrode array may provide the opportunity of structure preservation without discarding the advantages of a deep insertion in case the residual hearing gets lost.

This study is limited by the lack of assessing the postoperative insertion angle to prove the accuracy of the predicted insertion depth. However, studies assessing the prediction rate report only small prediction errors with a trend to overestimations of the insertion depth and larger errors with longer electrode arrays [30–33]. Furthermore, the preoperative CDL estimation was performed by a single investigator. However, the investigator is experienced interpreting temporal bone imaging and in one of our previous studies an excellent inter-rater reliability using the otosurgical planning software [15]. A further limitation interpreting the results is that the mathematical calculation for the visualization tool is based on the assumption that the electrode array is positioned along the lateral wall. However, the underlying calculation is based on the preoperative CDL output of the software that is calculated closer along the CDL_OC_. The CDL_OC_ is shorter compared to the CDL_LW_. Thus, the insertion depth may be biased and a discrepancy to linear estimation models occurs. There is evidence that this may lead to rather too short electrode array choices [15]. Consequently, there is a need for future studies assessing the accuracy of AID as well as LID prediction analyzing postoperative radiological imaging.

## Conclusion

The CC varies depending on the underlying CDL approximation, which critically influences electrode array choice. Based on the literature, we hypothesize that the non-linear method systematically overestimates the CC and may lead to rather too short electrode array choices. Longer flexible lateral wall electrode arrays of 28 mm and 31.5 mm are suitable to cover 70–80% of the CDL. In cases that exhibit more than one electrode array choice, the shorter electrode array may increase structure preservation while also increasing the chance of residual hearing preservation. This information may be of particular interest in areas with limited resources concerning presurgical planning software or high-resolution imaging. The results from this study add value to presurgical planning and may give recommendations on structure preserving electrode array choices. Future studies need to address the accuracy of the individual mathematical models.

## Data Availability

Not applicable.
